# Magnetic Nanoparticles in Biopolymer Fibers: Fabrication Techniques and Characterization Methods

**DOI:** 10.3390/polym16192805

**Published:** 2024-10-03

**Authors:** Mariana Bianchini Silva, Ulisses Oliveira Costa, Luiz Henrique Capparelli Mattoso, Sergio Neves Monteiro, Michele Lemos de Souza, Letícia Vitorazi

**Affiliations:** 1 Graduate Program in Metallurgical Engineering (PPGEM), EEIMVR, Fluminense Federal University, Avenida dos Trabalhadores, 420, Volta Redonda 27225-125, RJ, Brazil; maribianchinis@gmail.com (M.B.S.); uli_costa@id.uff.br (U.O.C.); 2 Graduate Program in Chemistry, Institute of Chemistry (IQ), University of Campinas, Rua Josué de Castro, s/n, Cidade Universitária, Campinas 13083-970, SP, Brazil; 3 Embrapa Instrumentation, National Laboratory of Nanotechnology for Agribusiness/LNNA, Rua 15 de Novembro, 1452, Centro, São Carlos 13560-970, SP, Brazil; luiz.mattoso@embrapa.br; 4 Department of Engineering and Materials Science, Military Institute of Engineering (IME), Rio de Janeiro 22290-270, RJ, Brazil; snevesmonteiro@gmail.com; 5 Department of Chemistry, University of Victoria, Victoria, BC V8P 5C2, Canada; michelesouzaca@gmail.com

**Keywords:** nanocomposite, biocompatible polymeric fiber, solution blow spinning, gelatin, poly(vinyl pyrrolidone), maghemite

## Abstract

Hybrid nanocomposites combining biopolymer fibers incorporated with nanoparticles (NPs) have received increasing attention due to their remarkable characteristics. Inorganic NPs are typically chosen for their properties, such as magnetism and thermal or electrical conductivity, for example. Meanwhile, the biopolymer fiber component is a backbone, and could act as a support structure for the NPs. This shift towards biopolymers over traditional synthetic polymers is motivated by their sustainability, compatibility with biological systems, non-toxic nature, and natural decomposition. This study employed the solution blow spinning (SBS) method to obtain a nanocomposite comprising poly(vinyl pyrrolidone), PVA, and gelatin biodegradable polymer fibers incorporated with magnetic iron oxide nanoparticles coated with poly(acrylic acid), PAA_2k_, coded as γ-Fe_2_O_3_-NPs-PAA_2k_. The fiber production process entailed a preliminary investigation to determine suitable solvents, polymer concentrations, and spinning parameters. γ-Fe_2_O_3_-NPs were synthesized via chemical co-precipitation as maghemite and coated with PAA_2k_ through the precipitation–redispersion protocol in order to prepare γ-Fe_2_O_3_-NPs-PAA_2k_. Biopolymeric fibers containing coated NPs with sub-micrometer diameters were obtained, with NP concentrations ranging from 1.0 to 1.7% wt. The synthesized NPs underwent characterization via dynamic light scattering, zeta potential analysis, and infrared spectroscopy, while the biopolymer fibers were characterized through scanning electron microscopy, infrared spectroscopy, and thermogravimetric analysis. Overall, this study demonstrates the successful implementation of SBS for producing biopolymeric fibers incorporating iron oxide NPs, where the amalgamation of materials demonstrated superior thermal behavior to the plain polymers. The thorough characterization of the NPs and fibers provided valuable insights into their properties, paving the way for their potential applications in various fields such as biomedical engineering, environmental remediation, and functional materials.

## 1. Introduction

Materials at the nanoscale, characterized by at least one dimension on the order of 10^−9^ m, exhibit distinct properties compared to bulk materials with similar composition [1,2,3]. This distinction arises from quantum effects, particularly pronounced as particle sizes approach the wavelength of electromagnetic radiation, leading to changes in material properties below a critical threshold and facilitating novel applications in nanomaterial development [4,5].

Nanoparticles (NPs), typically sized between 1 and 100 nm, represent a significant category of nanomaterials with diverse applications spanning from environmental remediation [6] to energy generation [7], electronics and sensors [8], cosmetics [9], and biomedicine [10,11,12]. Magnetic NPs offer unique capabilities, including manipulation via external magnetic fields for targeted drug delivery, localized tumor treatments, and tissue regeneration [12,13,14,15].

In this context, iron oxide nanoparticles (Fe_2_O_3_-NPs) have proven effective as fillers in synthesizing various nanomaterials with multiple functionalities in applications, such as biomedical components, personal protection, and electromagnetic radiation shielding, owing to their distinctive magnetic responsiveness [16,17]. Zhang et al. [18] suggest promising applications for iron oxide nanoparticles in polymer membranes for tissue engineering, particularly in skin regeneration. Their findings demonstrate that these membranes could be applied as suitable scaffolds for cell adhesion, as evidenced by in vitro cell culture, and exhibit lower cytotoxicity, highlighting their biomedical potential.

Moreover, the integration of metal nanoparticles (NPs) into polymer matrices has been recognized for enhancing the functional properties of these materials. Oun et al. [19] explored the synthesis, properties, and versatile applications of multifunctional nanocellulose-based hybrid nanomaterials incorporating metal or metal oxide nanoparticles such as Ag, ZnO, CuO, and Fe_3_O_4_-NPs, highlighting their engineering potential in fields like food packaging and smart fabrics. Additionally, researchers have explored the incorporation of metal oxide NPs into polymeric fibers for sensor technology, catalyst support, ultrafiltration systems, and industrial applications [20,21]. These fibers offer high surface area, strength, flexibility, and compatibility with biocompatible polymers [22,23,24]. Electrospinning remains a prevalent method for producing nano- and micropolymeric fibers. At the same time, emerging techniques like solution blow spinning (SBS) offer cost-effectiveness, high productivity, in situ utilization, and the potential for producing biopolymer fibers [25,26,27,28].

Both processes, electrospinning and solution blow spinning, can be applied to obtain nano- and microfibers from solutions employing polymers. However, the first process involves the application of a high voltage to promote fiber formation, while the second uses a high-speed air jet to promote fiber production. Comparative studies between the two methods have been presented to display the differences in the characteristics of the formed fibers [29,30]. However, solution blow spinning is reported as presenting a lower cost and greater scalability, while a high applied voltage is not necessary for the process.

Although gelatin has been used in the production of fibers via solution blow spinning [31,32], as well as in blends with PLA [33], and a mixture of iron particles was stabilized in hexafluoroisopropanol for the spinning process [34], the spinning of the PVP and gelatin blend has not been reported in the literature yet. Herein, we developed a new methodology for incorporating PAA_2k_-coated iron oxide nanoparticles into a new gelatin–PVP blend, which involved the stabilization of nanoparticles in the solvent mixture and careful incorporation into the polymeric solution. The novelty of this study is the use of PAA_2k_-coated nanoparticles in the production of fibers via SBS, as well as the use of the PVP/gelatin blend in spinning.

Incorporating NPs into polymeric fibers to create nanocomposite materials has garnered significant interest due to the synergistic combination of their unique properties [35]. This strategy offers notable advantages, as seen in examples such as introducing Ag-NPs into poly(vinyl alcohol) fibers, which endows them with potent antimicrobial capabilities [36,37,38]. Similarly, integrating zinc oxide NPs into poly(acrylonitrile) fibers elevates their photocatalytic degradation capacity, demonstrating the versatility and utility of this approach [39]. Given the promising attributes of nanomaterials, the versatility of polymeric fibers, and advancements in novel fiber production technologies, this study focused on implementing the SBS technique for developing biopolymeric fibers incorporated with PAA_2k_-coated γ-Fe_2_O_3_-NPs. Some coatings have been used to improve the stability of iron oxide nanoparticles in aqueous dispersion, driven by the use of the electrostatic adsorption of homo or block copolymers [40]. This protocol, called the precipitation–redispersion process, includes the use of poly(acrylic acid), PAA_2k_, which has been used to stabilize particles in a range of pH solutions, providing stabilization for cell culture medium [41], and has proven useful in different types of particles such as cerium and iron oxides [42].

## 2. Materials and Methods

### 2.1. Materials Preparation

**Figure 1:**
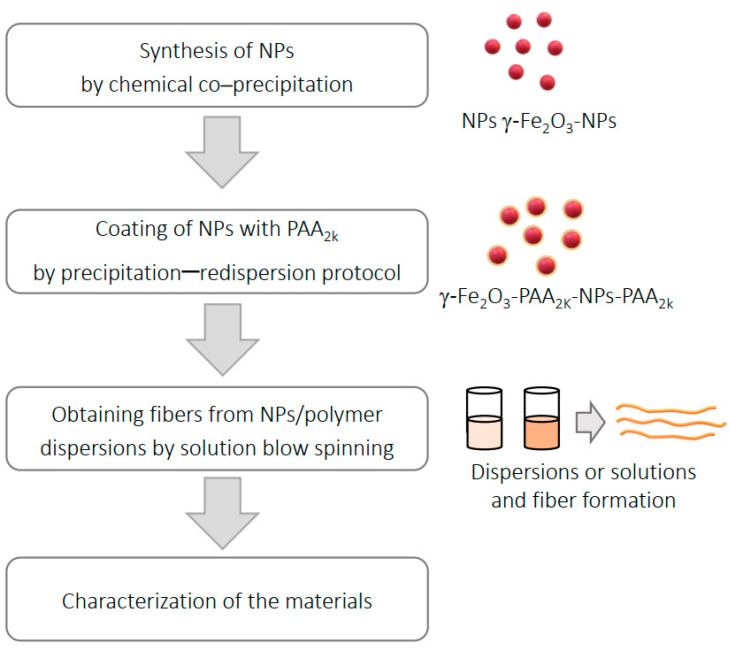
Scheme of the experimental steps.

The experimental procedure, outlined in Figure 1, began with the synthesis of iron oxide nanoparticles (γ-Fe_2_O_3_-NPs). Maghemite nanoparticles were selected due to their biocompatibility and superior chemical stability, as demonstrated by Sun et al. [43]. These nanoparticles (R = 0.25%) were then subjected to a coating process using poly(acrylic acid), yielding γ-Fe_2_O_3_ NPs coated with PAA_2k_ (γ-Fe_2_O_3_-NPs-PAA_2k_). Following this, biopolymeric fibers were fabricated via solution blow spinning, with different solvent systems being evaluated throughout the process. Finally, a blend containing bovine skin gelatin type B and poly(vinylpyrrolidone) (PVP) was obtained, and the γ-Fe_2_O_3_-NPs-PAA_2k_ were incorporated in the solution, ultimately resulting in iron oxide nanoparticles coated with PAA_2k_ embedded within the spun fibers. All the reagents and solvents used are listed in Supplementary Materials S2, Table S1.

#### 2.1.1. Synthesis of Iron Oxide (γ-Fe_2_O_3_) Nanoparticles (NPs)

The γ-Fe_2_O_3_-NPs were synthesized according to the Massart method [44]. A 50 mL aqueous solution was prepared from FeCl_3_·6H_2_O and FeCl_2_·4H_2_O, containing 0.13 mol L^−1^ total iron ion concentration, and a 0.5 ratio of Fe^2+^ to Fe^3+^. Sodium citrate (Na_3_C_6_H_5_O_7_·2H_2_O) was added to the solution at different concentrations, and the molar ratio between the citrate ions and Fe ions (R) was calculated according to Equation (1). The particles were synthesized with R values ranging from 0 to 3%.

(1) $$R=\frac{\left[{C_{6}H_{5}O_{7}}^{-}\right]}{\left[{\mathrm{Fe}}^{2+}\right]+[{\mathrm{Fe}}^{3+}]}\times \;100\%$$

where [C_6_H_5_O_7_], [Fe^2+^], and [Fe^3+^] correspond to the concentration of the citrate ions, Fe^2+^ ions, and Fe^3+^ ions, respectively, in mol L^−1^. Briefly, 3.30 mL of 28% NH_4_OH was slowly added to the Fe ion solution under magnetic stirring. A neodymium magnet was used to separate the precipitate from the supernatant, and the precipitate was washed twice with 25 mL of deionized water. Next, 5.30 mL of 2.0 mol L^−1^ HNO_3_ was added to the washed precipitate, and the solution was kept under stirring for 10 min. The precipitate was again separated by magnetic decantation, and the supernatant was removed. Finally, 7.90 mL of 0.34 mol L^−1^ Fe(NO_3_)_3_ was added to the precipitate, and the solution was heated at 90 °C for 30 min under magnetic stirring, leading to γ-Fe_2_O_3_ maghemite. The obtained precipitate was separated by magnetic decantation and dispersed in approximately 20 mL of deionized water. The pH ≤ 2 of the obtained dispersion containing citrate was verified using a properly calibrated pH meter. As shown in Figure S1, the qualitative magnetic properties of the γ-Fe_2_O_3_ nanoparticles can be observed through their interaction with a neodymium magnet, demonstrating their responsiveness to an external magnetic field. 

#### 2.1.2. Coating of Iron Oxide Nanoparticles

The coating was applied following the literature [40]. Approximately 2 g of PAA was dissolved in 1000 mL of deionized water acidified with HNO_3_ (pH ≈ 1.80). Using a separation funnel, a diluted solution of maghemite nanoparticles with R = 0.25% (10 mL of nanoparticles in 990 mL of acidified deionized water) was slowly added to the prepared PAA_2k_ solution while maintaining slow magnetic stirring. A neodymium magnet was used to remove the precipitate at the end of the addition, resulting in a concentrated nanoparticle dispersion (volume less than 10 mL). The pH of this solution was adjusted with NH_4_OH to approximately 9. The final solution was dialyzed in an alkaline medium (pH = 9) for 16 h to remove excess PAA_2k_.

#### 2.1.3. Polymeric Solutions and Nanoparticles Behavior in Dispersions

The stability of the synthesized γ-Fe_2_O_3_-NPs and coated γ-Fe_2_O_3_-NPs-PAA_2k_ NPs (R = 0.25%) was evaluated in different organic solvents: hexane, chloroform, acetone, isopropyl alcohol, ethyl alcohol, methyl alcohol, and acetic acid as in aqueous medium at different pH values. A determined amount of each solvent (1.00 mL) was placed in labeled glass vials, and up to 50 μL of either the synthesized or coated NPs in water dispersion were added. The behavior of the mixtures was observed for 30 min. The behavior in the aqueous solutions at different pH values is shown in Figure S2 in Supplementary Materials S3 where before coating (containing citrate), the particles are stable at ~pH ≤ 2 and after coating with PAA_2k_, the particles are stable at ~pH ≥ 4, and these characteristics indicate the success of the coating. After evaluating the stability of the nanoparticles, appropriate conditions were found regarding both stability for dispersion and ability for fiber formation, which was a polymeric mixture composed of 2.50% *w/v* gelatin and 6.00% *w/v* PVP in a mixture of 95% acetic acid (in water) and methanol (solvent ratio of 3:1 acetic acid to methanol). After that, five dispersions with incorporated NPs were prepared, considering nanoparticle mass calculated relative to the total polymer mass: 0; 1.00; 1.25; 1.50; and 1.70% wt. of γ-Fe_2_O_3_-NPs-PAA_2k_.

### 2.2. Obtaining Fibers Incorporated with NPs

Several polymeric solutions were prepared for testing in the spinning system to evaluate effective fiber formation and solvent evaporation, the quality of formed fibers, and processing parameters for the subsequent NPs incorporation, as described in Supplementary Materials S4 (Table S2 and Figure S3). Table 1 presents the processing parameters used for spinning the five polymeric solutions with incorporated NPs, including solution feeding rate, air pressure, the distance between the needle and collector, and the environmental conditions (relative humidity and temperature) for each solution. Each solution was prepared with a total volume of 5.00 mL, and the spinning process took an average of 30 to 40 min, yielding sufficient material for the subsequent analyses. The compositions and preparation methods of the solutions used in the SBS process are in Supplementary Materials S4.

All the samples were collected on aluminum foil positioned over a cylindrical rotary collector operating at a rotation speed of 180 rpm and on the foil positioned behind the rotary collector (static collector).

### 2.3. Characterization

#### 2.3.1. Analysis by Dynamic Light Scattering and Zeta Potential

The synthesized γ-Fe_2_O_3_-NPs and coated γ-Fe_2_O_3_-NPs-PAA_2k_ (R = 0.25%) were analyzed by dynamic and electrophoretic light scattering (DLS and ELS) to determine their hydrodynamic diameter and zeta potential. All measurements were performed using the Litesizer 500 or 700 particle analyzer (Anton Paar, Graz, Austria) at 25 °C. The dynamic light scattering analysis was performed on polystyrene cuvettes using the automatic mode to select the angle and 30 s as the equilibrium time. The zeta potential was determined through Smoluchowski approximation using Ômega polystyrene cuvette. For both measurements and all the samples evaluated, 10 µL of NP dispersion from the synthesis was used in 1 mL of deionized water at pH = 2 or pH 9 for the coated particle γ-Fe_2_O_3_-NPs-PAA_2k_.

#### 2.3.2. Infrared Spectroscopy (FTIR)

The infrared spectra of the uncoated NP, the polymers polyvinylpyrrolidone (PVP) and gelatin, and the produced fibers incorporated or not with NPs were recorded using the FT/IR 4700 spectrometer (JASCO, Tsukuba, Japan) using potassium bromide (KBr) pellets. Scans were performed from 400 to 4000 cm^−1^ for each sample with a resolution of 4 cm^−1^. The data were recorded regarding transmittance using the Spectra Manager^TM^ software, version 2 (Jasco).

#### 2.3.3. Scanning Electron Microscopy (SEM)

SEM images of the polymeric fibers were obtained using the Carl Zeiss EVO MA10 Scanning Electron Microscope (Carl Zeiss Pvt. Ltd., Baden-Württemberg, Germany). The diameter of the fibers was determined through image processing using the ImageJ software, version 1.51 (National Institute of Health, Bethesda, MD, USA) utilizing images with magnification between 1000 and 2000 times and at least 150 diameter measurements.

#### 2.3.4. Thermogravimetric Analysis (TGA)

The curves of two fiber samples, with 0 and 1.70% of NPs, were obtained using the Q500 analyzer (Thermal Analysis Instruments, New Castle, NJ, USA). The analyses were conducted under an inert atmosphere with nitrogen gas at 40 and 60 mL/min flow rates in the balance and sample, respectively, with a heating ramp from 25 to 550 °C at 10 °C/min. Samples weighing between 10 and 12 mg were used for the analyses. The collected data were processed using the TA Universal Analysis 2000 software.

#### 2.3.5. Transmission Electron Microscopy (TEM)

The morphology and crystal structure of the GNPs were examined using high-resolution transmission electron microscopy (HR-TEM) and selected area electron diffraction (SAED). These analyses were performed on a JEOL 2100 F electron microscope (Tokyo, Japan) equipped with a CMOS camera and operated at an acceleration voltage of 200 kV.

#### 2.3.6. X-ray Diffraction Analysis (XRD)

XRD analyses were conducted to assess the amorphous, crystalline, or semi-crystalline nature of the samples. The measurements were carried out using a Panalytical X’Pert Pro diffractometer (Malvern Panalytical Ltd, Malvern, Worcestershire, UK) equipped with a cobalt (Co) anode and a scintillation counter detector (NaI). The instrument operated at 40 mA and 40 kV. The scanning range for the analysis was set from 10° to 80°, utilizing a θ–2θ configuration.

## 3. Results

### 3.1. Nanoparticles and Individual Polymers Characterization

#### 3.1.1. Size and Surface Charge of Particles

The hydrodynamic diameter and zeta potential of the synthesized γ-Fe_2_O_3_-NPs were determined through light scattering and are summarized in Table 2.

The hydrodynamic diameter and zeta potential values were recorded for the particles synthesized with varying amounts of sodium citrate for comparison purposes. The hydrodynamic diameter varied from 48 to 124 nm, and the zeta potential ranged from 21.62 to 32.8 mV. Across all the examined samples, the zeta potential exhibited absolute values hovering around +30 mV, indicating significant repulsion between particles and a propensity to maintain dispersion in highly acidic environments (pH ≈ 2) [45,46]. In all the cases, a positive zeta potential was observed, likely due to the presence of nitrate ions (NO_3_^−^) originating from nitric acid alongside citrate ions adsorbed onto the surface [47]. Additionally, it was observed that the increase in the sodium citrate concentration did not significantly impact the particle surface charge. However, the addition of the citrate ions in the synthesis influenced the particle size, which increased considerably with higher amounts of citrate.

A qualitative test revealed that the NPs coagulate and precipitate when the pH increases up to 3.5 depending on the size of the nanoparticle, as the literature describes [48]. Hence, a precipitation–redispersion procedure was conducted to coat the synthesized NPs with a PAA_2k_ polymer [49], enhancing particle stability in complex media with polymers and organic solvents. Then, considering particle size (smaller) and surface charge (more positive) from Table 2, the γ-Fe_2_O_3_-NPs sample with R = 0.25% was chosen to be coated with PAA_2k_.

During the coating of iron oxide nanoparticle procedure, the slow addition of the diluted NP solution to the polymer solution at pH = 2 induced macroscopic precipitation. Adding NH_4_OH to the isolated precipitate resulted in the spontaneous redispersion of the NPs, yielding a concentrated solution of individually PAA_2k_-coated particles stable within a pH range of 4 to 10, with ammonium ions (NH_4_^+^) potentially acting as counterions to acrylate ions (CH_2_CHOO^−^) [47,48,49]. The final dialysis step removes unreacted polymeric chains from the solution [49]. This precipitation–redispersion method is a widely studied and applied procedure for NP coating. It yields highly stable particles with a negative surface charge due to the polyanionic characteristic of the PAA polymer [40,47,49,50]. In this study, the coated NP (R = 0.25 from Table 2, γ-Fe_2_O_3_-NPs-PAA_2k_, pH 9) presented a hydrodynamic diameter of 54.3 ± 0.5 nm and zeta potential of −55.8 ± 5.6 mV. The size of the coated nanoparticle was slightly larger than that of the uncoated particle. This aligns with expectations since there is a polymer layer on the particle’s surface. Most evidently, a change in zeta potential was observed. Coating with PAA leads to obtaining a material with negative potential, indicating the anionic polyelectrolyte PAA’s adsorption at the nanoparticle’s surface.

#### 3.1.2. Infrared Spectroscopy (FTIR) of the NPs and Individual Polymers

**Figure 2:**
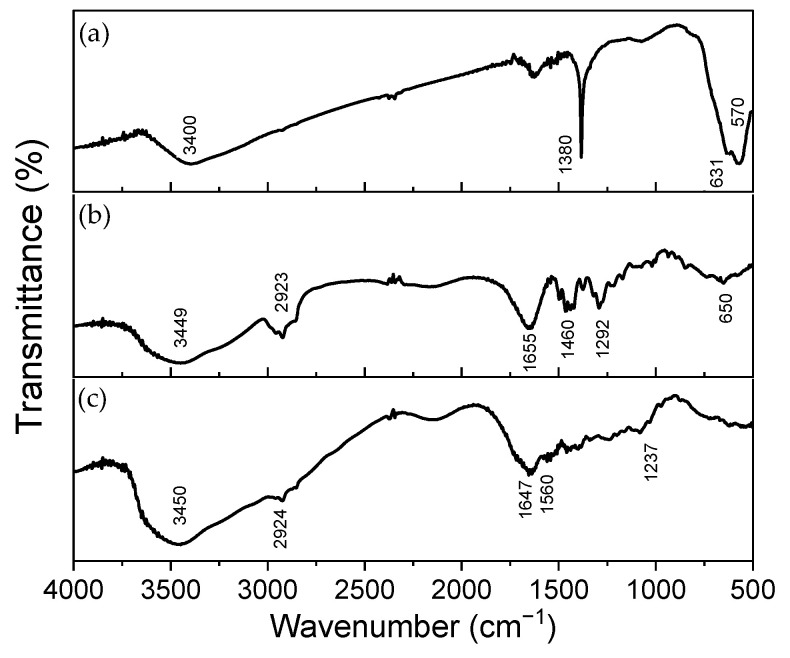
Infrared spectra of (**a**) γ-Fe_2_O_3_-NPs, PVP (**b**) and gelatin polymer (**c**) in KBr pellet.

Figure 2 presents the FTIR data, and the main bands associated with the chemical groups are in Table 3. The FTIR spectra of γ-Fe_2_O_3_-NPs (Figure 2a) were acquired and their significant bands assigned, as well as the spectra for the precursor polymers (Figure 2b,c). When analyzing the NPs FTIR spectrum, significant bands are observed at 570 cm^−1^ and 631 cm^−1^, associated with the Fe-O bond vibration [51,52,53], as well as a narrow band at 1380 cm^−1^ and a broad band around 3400 cm^−1^, related to the angular deformation of O-H and the stretching of the O-H bond of hydroxyl groups (-OH) due to the presence of adsorbed water [53,54]. γ-Fe_2_O_3_ was synthesized with citrate and citrate has bands at 1380 cm^−1^ and in approximately 1585 cm^−1^ and 1622 cm^−1^. Since the FTIR technique is concentration-dependent, we can infer that the amount of citrate is significantly low to be identified in the FTIR. Then, the main technique supporting the coating of the NPs before treatment with PAA is the zeta potential. It is important also to note the difference between the spectrum obtained for the synthesized nanoparticle and those reported in the literature for magnetite and hematite. The Fe-O bond vibration for magnetite appears at the bands at 580 and 400 cm^−1^ [55,56], and the vibration of this bond for hematite appears around 560 and 470 cm^−1^ [53].

Among iron oxide polymorphs, Fe_3_O_4_ and Fe_2_O_3_ are the most explored due to their high abundance and versatility [57]. Magnetite, Fe_3_O_4_, is the most magnetic naturally occurring mineral on earth, and it presents a face-centered cubic spinel crystal structure at room temperature, where Fe^2+^ and Fe^3+^ cations are present. Stoichiometric magnetite presents a Fe^2+^ and Fe^3+^ ratio of 1:2. On the other hand, the Fe_2_O_3_ form of iron oxide exists in four polymorphs, where maghemite (γ-Fe_2_O_3_) and hematite (α-Fe_2_O_3_) are the most explored. Magnetite (Fe_3_O_4_) can oxidize into the maghemite phase by natural or synthetic processes, where all the Fe^2+^ ions are converted into Fe^3+^ ions. Maghemite is transformed into hematite through a high-temperature process, which is required because their crystal structures are different. While maghemite presents a cubic spinel structure, hematite crystallizes in the corundum structure. Although maghemite and hematite present similar chemical composition, where only Fe^3+^ ions are present, their different crystal structures grant different properties, such as their magnetic behavior: maghemite is ferrimagnetic at room temperature, while hematite is weakly ferromagnetic at room temperature and presents paramagnetic behavior only at elevated temperatures [57,58].

FTIR spectra were also recorded for the constituent polymers used in the fibers. The spectrum for the PVP polymer is shown in Figure 2b. It highlights bands at 1655 cm^−1^, associated with the stretching of the C=O bond in the ring; at 1292 cm^−1^, related to the vibration of the C-N bond; and bands at 1455 and 1422 cm^−1^, characteristic of the pyrrolidinyl group (derived from pyrrolidine, a cyclic amine with a ring containing four carbon atoms) [59]. Additionally, a broad band at 3449 cm^−1^, associated with the stretching of the O-H bond of hydroxyl groups from adsorbed water, and bands at 2923, 1460, and 650 cm^−1^, related to the stretching of the C-H bond in the CH_2_ group, angular deformation in the CH_2_ groups, and angular deformation of C-H, respectively, can be highlighted [59,60].

Unlike PVP, gelatin is a polymer composed of a heterogeneous mixture of polypeptide fragments. Its infrared spectrum, shown in Figure 2c, provides information about the vibration of peptide bonds and the protein’s secondary structure. It exhibits up to nine characteristic bands, although interpreting all of them is highly complex and impractical [61].

The Amide I band corresponds to the stretching of the C=O bonds in peptide linkages, while the Amide II and Amide III bands are both related to the stretching of the C-N bonds and the angular deformation of the N-H bonds in the plane [61]. In the higher wavenumber region, the Amide A and B bands are found; the former corresponds to the stretching of the N-H bond coupled with hydrogen bonds, and the latter to the asymmetric stretching of the C-H bonds in sp^2^ carbons [60,61]. Additionally, the presence of adsorbed water in the material should be considered. Thus, the broad band located around 3450 cm^−1^ may contribute to the stretching of O-H bonds. The experimental spectrum obtained for gelatin is consistent with what is reported in the literature [61].

#### 3.1.3. Structural Characterization of γ-Fe_2_O_3_-NPs-PAA_2k_

**Figure 3:**
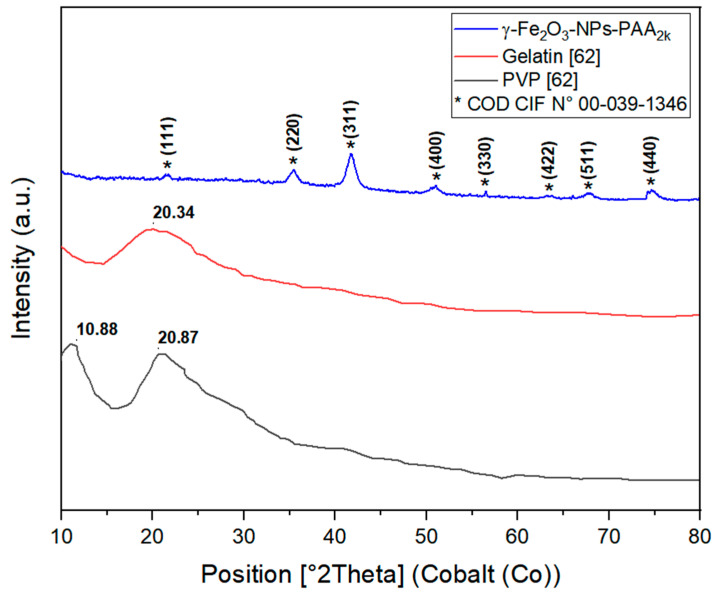
XRD diffractogram for PVP, gelatin and γ-Fe_2_O-NPs-PAA_2k_.

The XRD profiles of pure gelatin, PVP, and γ-Fe_2_O_3_-NPs-PAA_2k_ were analyzed to determine their amorphous, crystalline, or semi-crystalline nature. As illustrated in Figure 3, there is a significant change in the XRD patterns of the blend compared to those of pure gelatin and PVP. The XRD scan of pure gelatin shows a broad diffraction peak at approximately 2θ = 20.34°, indicating its amorphous nature. The XRD pattern of pure PVP reveals two distinct peaks: a sharper, more intense peak at 2θ = 10.88° and a broader, less intense peak at 2θ = 20.87° [61].

Figure 3 provides a detailed visualization of these diffraction peaks and their corresponding crystallographic planes. For the γ-Fe_2_O_3_-NPs-PAA_2k_, the XRD diffractogram displays eight prominent peaks at 2θ values of 21.55°, 35.41°, 41.73°, 50.87°, 56.50°, 63.34°, 67.80°, and 74.67°, which correspond to the (111), (220), (311), (400), (331), (422), (511), and (440) crystallographic planes, respectively. These results match well with the standard diffraction data for γ-Fe_2_O_3_ with a cubic crystal system, P4132 space group, and lattice parameter of 0.83515 nm, as referenced by the COD CIF No. 00-039-1346.

#### 3.1.4. Morphological Analysis of γ-Fe_2_O_3_-NPs-PAA_2k_

**Figure 4:**
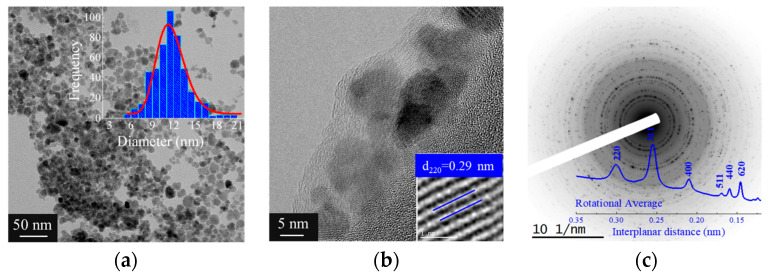
TEM analysis of coated iron oxide nanoparticles (γ-Fe_2_O_3_-NPs-PAA_2k_): (**a**) TEM image of nanoparticles and size distribution of coated nanoparticles, (**b**) detailed TEM of coated nanoparticles and calculated interplanar distance for (220) plane and (**c**) X-ray diffraction pattern of coated nanoparticles.

Figure 4 presents the TEM analysis of iron oxide nanoparticles coated within fibers. The nanoparticles appear well dispersed without noticeable agglomeration, with an average diameter of approximately 12 nm, as shown by the size distribution in Figure 4a. The diameter determined by TEM is smaller than the hydrodynamic diameter obtained from dynamic light scattering (DLS), as expected.

Figure 4b presents a higher-magnification TEM image highlighting the detailed structure of the coated nanoparticles. In this image, the morphological features of the nanoparticles are clearly visible, including the calculation of the interplanar distance corresponding to the (220) plane. The observed interplanar distance reinforces the identification of the crystalline phase and provides visual confirmation of the atomic spacing, in agreement with the X-ray diffraction (XRD) data.

Figure 4c shows the XRD pattern of the coated nanoparticles, indicating the presence of crystalline phases in the material. The diffraction peak positions are consistent with the expected crystallographic planes of maghemite (Fe_2_O_3_), and the analysis of the pattern suggests good crystallinity, complementing the TEM observations.

The TEM images demonstrate the incorporation of the nanoparticles within the fiber matrix, with magnified regions provided in Figure 4b,c offering a more detailed view of the particle morphology and crystalline structure. A homogeneous distribution of the nanoparticles within the matrix is observed, and notably, the preservation of the structural features in the magnified areas suggests a favorable interaction between the matrix and the coated particles.

### 3.2. Nanocomposite Polymer Fiber Materials Characterization

#### 3.2.1. Obtaining Polymeric Fibers

The procedure for incorporating NPs into the polymer solution and, consequently, into the fibers involved selecting suitable solvents and polymers, determining the polymer concentration, and combining and proportioning solvents for fiber production. To adjust the solvent, the compatibility and stability of the synthesized and coated particles were evaluated in various organic solvents. The coated NPs were compatible with polar solvents (alcohols, acetone, and acetic acid) and phase separation in less polar solvents (hexane and chloroform). The as-synthesized NPs were stable in highly acidic conditions, with a pH of around two, and did not exhibit stability in organic solvents. Thus, coating with PAA_2k_ was a stabilization strategy for incorporating the NPs into the polymer solution.

After identifying the suitable solvents compatible with the coated NPs, various polymer mixtures were assessed to form fibers from gelatin and PVP at different concentrations and solvent ratios. One mixture, comprising 2.5% *w/v* gelatin and 6.0% *w/v* PVP in 95% acetic acid and methanol at a 3:1 ratio, emerged as the best option for producing fibers with incorporated γ-Fe_2_O_3_-NPs-PAA_2k_. This conclusion was drawn based on the solution blow spinning process’s efficiency, the quality of the fibers produced, and the ease of NP incorporation into the polymer solution. Acetic acid was chosen for its ability to dissolve both polymers, supplemented with a small amount of water to dissolve the gelatin completely. Methanol was selected for its superior ability to dissolve PVP and its high vapor pressure, ensuring suitable volatility when combined with acetic acid. Spinning tests and parameter adjustments determined the optimal solvent proportion. The experimental observations revealed that this mixture provided the most effective fiber formation, demonstrating superior spinning efficiency, high-quality fibers, and minimal defects, outperforming the other tested solutions, which faced issues like unstable spinning cones, drops on the collector, and fibers with beads.

**Figure 5:**
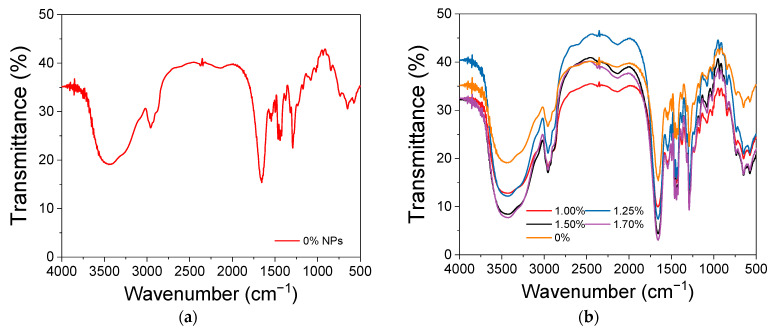
Infrared spectra for (**a**) PVP with gelatin fiber without NPs incorporation and (**b**) PVP with gelatin fibers with increasing NPs concentration.

Consequently, NPs were introduced into the polymer solution in limited quantities, with a maximum concentration of 1.70% *w/w* (relative to the total polymer mass), equivalent to 1.47 g/L of NPs. However, increasing the NP dosage led to the formation of a turbid solution, indicating material precipitation and the disruption of NP homogeneity within the polymer solution. This emphasizes the importance of precise NP addition to maintain solution stability and ensure uniform distribution, which is essential for successful NP integration into the polymer matrix. Figure 5 shows the IR spectra for all the analyzed fibers without incorporated nanoparticles (Figure 5a) or containing particles (Figure 5b).

The observations reveal that the spectra of the five produced fibers (Figure 5 exhibit similar shapes and bands among themselves, indicating that the overall composition of the fibers remains unchanged despite being derived from separately prepared solutions. Significant bands are observed around 3450, 2950, 2920 (shoulder-shaped), and 1660 cm^−1^, consistent with what was observed in the precursor polymeric materials (Figure 2b,c). Furthermore, the presence of NPs in the fibers could not be identified by FTIR due to their low concentration relative to the total material. Transmission electron microscopy is suggested for the precise identification of NPs in the produced fibers.

#### 3.2.2. Structural Characterization of the Nanocomposite Fibers

**Figure 6:**
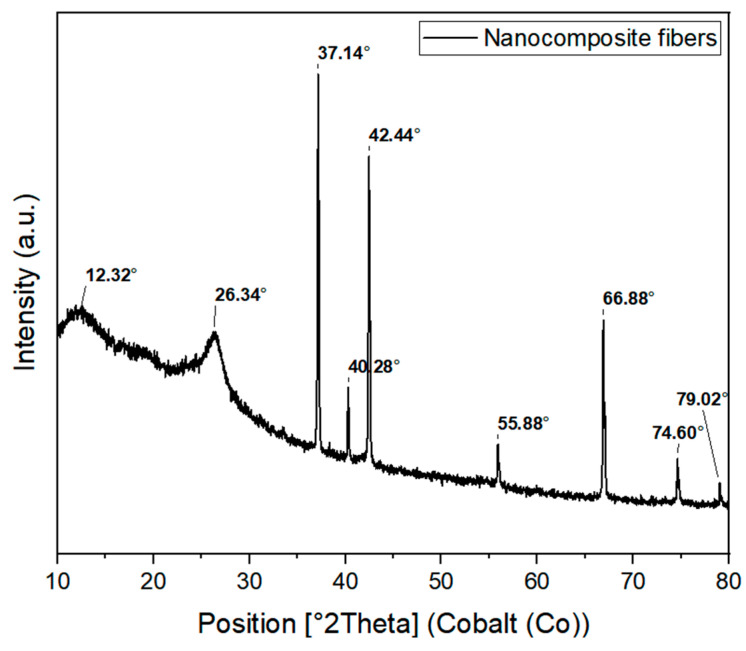
XRD diffractogram of nanocomposite fibers with γ-Fe_2_O_3_-NPs.

Upon incorporation into the nanocomposite fibers, two prominent peaks appeared in the XRD diffractogram at 2θ = 12.32° and 2θ = 26.34°. Notably, these peaks are shifted to higher angles compared to those of the pristine polymers, Figure 6, suggesting potential interactions between the γ-Fe_2_O_3_ nanoparticles (NPs) and the gelatin–PVP polymer blend during the spinning process [62]. This shift indicates the formation of new crystalline structures or rearrangements within the polymer matrix, influenced by the presence of the NPs. The peak at 40.28° corresponds to the (111) plane of the cubic Fe-Si wafer substrate, as identified by reference pattern No. 03-065-1835.

Further support for these interactions is provided by the FTIR analysis. Although the FTIR spectra (Figure 5b) of the fibers containing NPs exhibit similar shapes and bands to those of the precursor polymers (Figure 2b,c), indicating that the overall chemical composition of the fibers remains largely unchanged, the XRD results reveal that the incorporation of γ-Fe_2_O_3_ NPs induces structural modifications at the crystalline level.

In conclusion, the combined XRD and FTIR analyses demonstrate that while the γ-Fe_2_O_3_ NPs are successfully incorporated into the polymer matrix, leading to structural modifications as evidenced by the XRD peak shifts, these changes do not significantly alter the overall chemical composition of the fibers, as confirmed by FTIR. This correlation between the structural and chemical analyses offers a comprehensive understanding of the nanocomposite’s characteristics.

#### 3.2.3. Morphological Analysis of Polymer Nanocomposite Fibers

**Figure 7:**
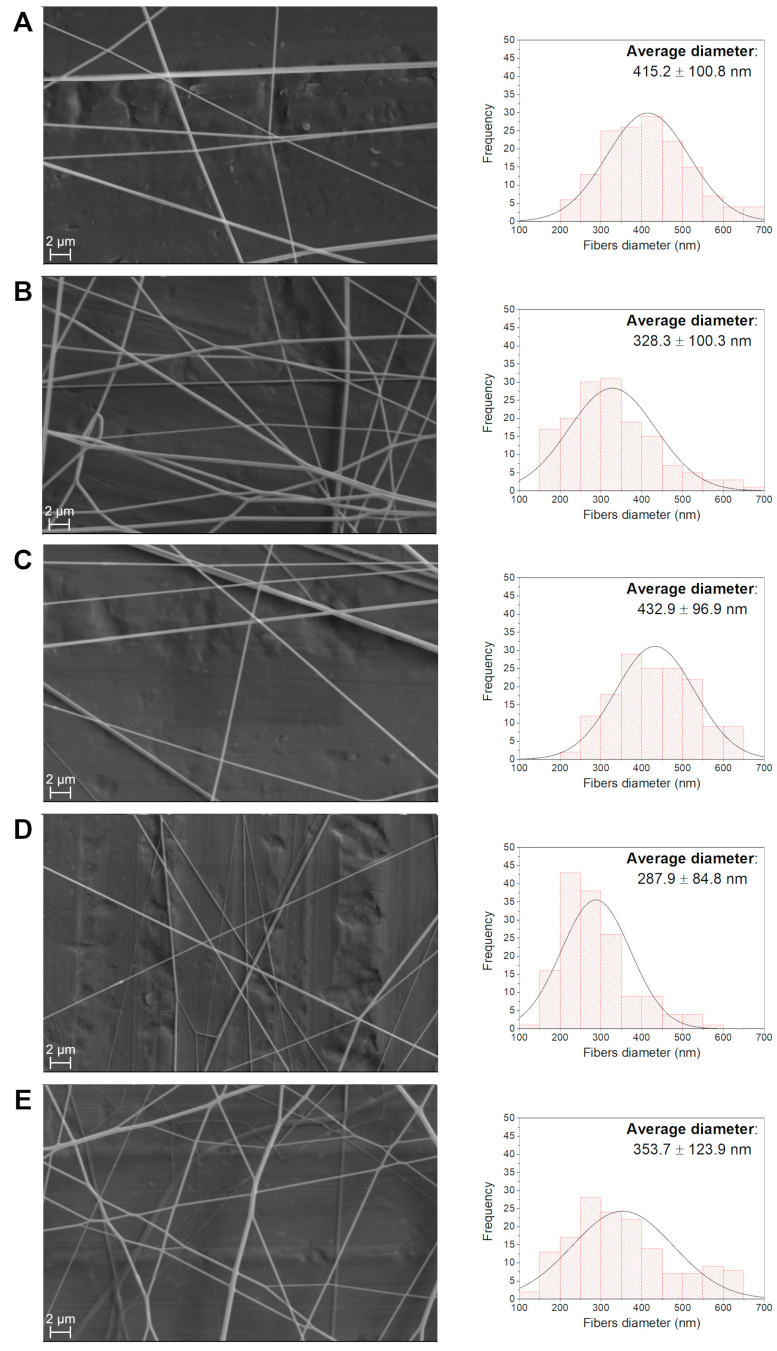
SEM images at 3000× magnification and fiber size distribution for produced fibers with (**A**) 0% incorporated NPs, (**B**) 1.00% NPs, (**C**) 1.25% NPs, (**D**) 1.50% NPs and (**E**) 1.70% NPs.

SEM images were used to determine the average diameter of each produced fiber. The obtained sizes are summarized in Table 4. Figure 7 presents the microscopy images recorded with a 3000× magnification for each sample and their corresponding size distribution curves. Sub-micrometer scale fibers ranging from 288 to 415 nm (diameter) were produced. It was observed that the incorporation of NPs does not uniformly affect the diameter of the fibers, meaning that adding a larger quantity of particles did not increase or decrease fiber size. However, NP incorporation significantly affected the uniformity of the size distribution.

**Figure 8:**
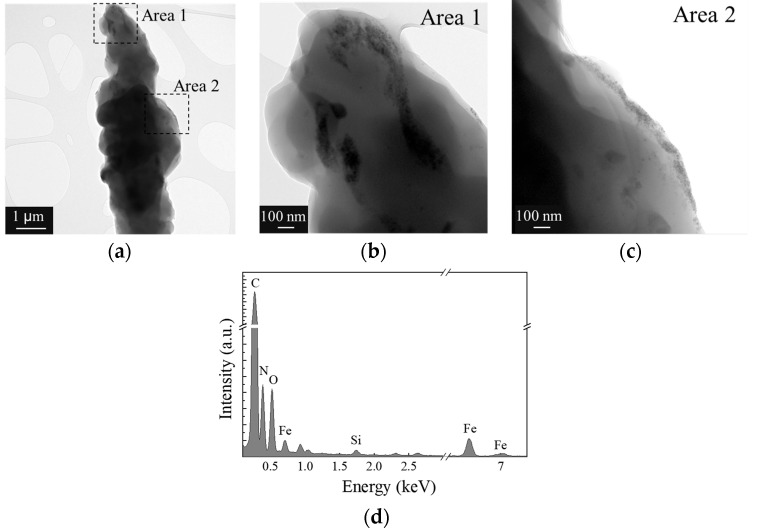
TEM images of the nanocomposite fibers: (**a**) fiber with 1.70% (weight) incorporated nanoparticles, where two different regions are highlighted, (**b**) detailed image of area 1 highlighted, (**c**) detailed image of area 2 highlighted and (**d**) energy dispersive X-ray spectrum of fibers with incorporated nanoparticles.

Furthermore, the X-ray diffraction pattern confirms the crystalline phases of the nanoparticles within the fiber matrix. In Figure 8, the TEM images reveal the successful incorporation of nanoparticles within the fibers, evidenced by the two distinct regions highlighted in the fiber. Figure 8b,c provide the magnified views of area 1 and area 2, respectively, which display more detailed structures of the embedded nanoparticles. The TEM images clearly depict the presence of the nanoparticles, offering insight into their spatial distribution and interaction with the polymeric fiber matrix.

Additionally, the EDS analysis in Figure 8d provides the elemental composition of the fibers. The spectrum shows significant peaks for carbon, nitrogen, and oxygen, which are expected due to the polymeric nature of the fiber material. The presence of iron, detected in smaller but prominent amounts, further confirms the successful incorporation of the Fe_2_O_3_ nanoparticles within the fiber.

The TEM image presented in Figure 8a shows the fiber containing 1.70 wt% of the incorporated nanoparticles, with areas 1 and 2 specifically highlighted for further examination. The detailed images in Figure 8b,c provide a closer look at the nanoparticle distribution and morphology within these regions, while the EDS spectrum in Figure 8d confirms the elemental composition of the fibers, further supporting the presence of the nanoparticles.

#### 3.2.4. Thermal Analysis of the Polymer Nanocomposite Fibers 

TGA was conducted to assess the thermal behavior of the produced fibers and the influence of the iron oxide NP addition. Although the fibers with 1.5% NP exhibited the smallest diameters, the sample containing 1.7% NP was selected for TGA to maximize the detection of the nanoparticles’ influence on the thermal properties. Figure 5 displays the mass loss curves as a function of temperature (TG) and the first derivative of mass loss as a function of temperature (DTG) for two fiber samples, with minimum and maximum amounts of NPs, while Table 5 summarizes the TGA data.

**Figure 9:**
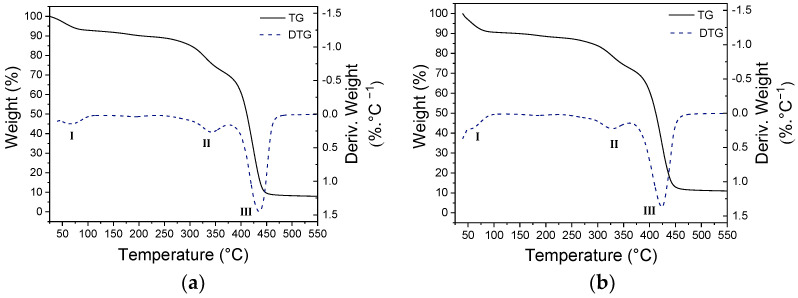
Mass loss curves as a function of temperature (solid line) and the corresponding first derivative (dashed line) for TGA recorded in an inert atmosphere of fibers with (**a**) 0% NPs and (**b**) 1.70% NPs.

The thermal degradation of pure gelatin and PVP polymers occurs from 250 and 330 °C, respectively [63]. With the combination of both polymers, mass loss occurs in three distinct stages, as observed in the DTG curves in Figure 9: the first stage, (I), corresponds to water evaporation and it extends to approximately 100 °C; the second stage, (II), with a derivative peak around 330 °C, corresponds to gelatin degradation; and the last stage, (III), with a derivative peak near 420 °C, corresponds to PVP degradation. The thermal behavior of the material was minimally influenced by the addition of NPs: the T_onset_ and T_D_ values are similar, and the mass loss was slightly lower in each stage for the sample with NPs. However, it can be observed that the sample with incorporated NPs exhibits higher residual mass: at 550 °C, the sample without NPs showed 7.95% residue, while the sample with NPs showed 10.92%, indicating a difference of almost 3%; this result indicates the presence of inorganic material that persists after the degradation of the polymeric matrix.

## 4. Conclusions

In this study, we successfully implemented the solution blow spinning system for the fabrication of polymeric fibers incorporating magnetic γ-Fe_2_O_3_ NPs coated with PAA_2k_ using five distinct concentrations of these NPs. The synthesis of the maghemite nanoparticles, confirmed by the XRD and TEM analyses, showed that the nanoparticles had the desired crystalline structure and nanoscale size. The dynamic light scattering further validated the nanoscale dimensions, and the zeta potential confirmed the suitability of the poly(acrylic acid) coating for stable nanoparticle dispersion in polymer solutions.

The production of polymeric fibers with gelatin and PVP, incorporating the γ-Fe_2_O_3_-NPs-PAA_2k_, resulted in fibers with submicron diameters and homogeneous chemical composition, as observed by SEM and FTIR. The TGA revealed enhanced thermal properties in the fibers with incorporated nanoparticles, particularly an increased residual mass, confirming the presence of the inorganic material. Furthermore, the fibers exhibited good thermal stability, making them suitable for potential applications where thermal resistance is a critical requirement.

These results confirm that the integration of γ-Fe_2_O_3_-NPs-PAA_2k_ into polymeric fibers using the solution blow spinning technique is an effective approach for producing composite fibers with tailored thermal and structural properties.

## Figures and Tables

**Table 1:** Parameters used in solution blow spinning to obtain polymeric fibers.

NP Content (%)	Feeding Rate (mL/h)	Air Pressure (kg/cm^2^)	Needle–Collector Distance (cm)	Relative Humidity (% H_2_O)	Temperature (°C)
0	1.70	1.00	35	50	23.2
1.00	1.80	1.00	30	57	20.9
1.25	1.80	1.00	35	51	22.5
1.50	1.80	1.00	25	58	21.0
1.70	1.80	1.00	28	55	21.2

**Table 2:** Experimentally obtained hydrodynamic diameter and zeta potential values for nanoparticles synthesized with increasing amounts of sodium citrate, at pH = 2.

R (%)	Hydrodynamic Diameter (nm)	Zeta Potential (mV)
0	93 ± 11	+26.3 ± 1.3
0.25	54 ± 1	+32.2 ± 1.1
0.50	73 ± 1	+29.5 ± 1.4
0.75	60 ± 7	+32.8 ± 1.6
1.00	48 ± 1	+21.6 ± 1.1
2.00	89 ± 5	+30.4 ± 1.4
3.00	83 ± 5	+34.4 ± 1.5
3.50	124 ± 4	+30.9 ± 1.3

**Table 3:** The main bands and chemical groups of the infrared spectra of γ-Fe_2_O_3_-NPs, PVP and gelatin polymer from the data are presented in Figure 2.

γ-Fe_2_O_3_-NPs		PVP Polymer		Gelatin Polymer	
Band (cm^−1^)	Chemical Group	Band (cm^−1^)	Chemical Group	Band (cm^−1^)	Chemical Group
3400	Stretching O-H	3449	Stretching O-H	3450	Amide A
1380	Angular deformation H-OH	2923	Stretching C-H of CH_2_ group	2924	Amide B
631	Vibration Fe-O	1655	Stretching C=O of the ring	1647	Amide I
570	Vibration Fe-O	1460	Angular deformation CH_2_	1560	Amide II
-	-	1292	Stretching C-N	1237	Amide III
-	-	650	Angular deformation C-H	-	-

**Table 4:** Average diameters of the fibers and associated deviations obtained by applying a normal distribution to the analyzed histograms.

NP Content (%)	Average Fiber Diameter (nm)	Standard Deviation (nm)
0	415.2	100.8
1.00	328.3	106.5
1.25	432.9	96.9
1.50	287.9	84.8
1.70	353.7	123.9

**Table 5:** Summarized thermogravimetric analysis data for degradation stages II and III, including T_onset_, mass loss (Δ_m_) and temperature at the maximum mass loss rate (T_D_).

	II			III		
	T_onset_ (°C)	Δ_m_ (%)	T_D_ (°C)	T_onset_ (°C)	Δ_m_ (%)	T_D_ (°C)
**0%**	306	13.30	329	402	65.25	423
**1.70%**	304	11.60	327	401	62.16	424

## Data Availability

All the data underlying the results are available as part of the article and no additional source data are required.

## Supplementary Materials

The following supporting information can be downloaded at: https://www.mdpi.com/article/10.3390/polym16192805/s1. S1—Qualitative demonstration of the magnetic responsiveness of γ-Fe_2_O_3_-NPs, S2—List of all reagents and solvents used in studies, S3—The behavior of nanoparticles as a function of the pH of the medium, S4—Composition and preparation methods of solutions used in the spinning process.

## References

[1] Saleh T.A. (2020). Nanomaterials: Classification, properties and environmental toxicities. Environ. Technol. Innov..

[2] Joudeh N., Linke D. (2022). Nanoparticle classification, physicochemical properties, characterization and applications: A comprehensive review for biologists. J. Nanobiotechnol..

[3] Prasad R.D., Prasad R.S., Prasad R.B., Prasad S.R., Singha S.B., Singha D., Navathe G.J. (2024). A review on modern characterization techniques for analysis of nanomaterials and biomaterials. ES Energy Environ..

[4] Ulusoy U. (2023). A review of particle shape effects on material properties for various engineering applications: From macro to nanoscale. Minerals.

[5] Idumah C.I., Obele C.M. (2021). Understanding interfacial influence on properties of polymer nanocomposites. Surf. Interfaces.

[6] Rafeeq H., Hussain A., Ambreen A., Waqas M., Bilal M., Iqbal H.M. (2022). Functionalized nanoparticles and their environmental remediation potential: A review. J. Nanostruct. Chem..

[7] Kumar A., Choudhary P., Kumar A., Camargo P.H., Krishnan V. (2022). Recent advances in plasmonic photocatalysis based on TiO_2_ and noble metal nanoparticles for energy conversion, environmental remediation and organic synthesis. Small.

[8] Cheng H.W., Yan S., Shang G., Wang S., Zhong C.J. (2021). Strain sensors fabricated by surface assembly of nanoparticles. Biosens. Bioelectron..

[9] Sharma A., Agarwal P., Sebghatollahi Z., Mahato N. (2023). Functional nanostructured materials in the cosmetics industry: A review. ChemEngineering.

[10] Lu C.H., Hsiao J.K. (2023). Diagnostic and therapeutic roles of iron oxide nanoparticles in biomedicine. Tzu Chi Med. J..

[11] MubarakAli D., Kim H., Venkatesh P.S., Kim J.W., Lee S.Y. (2023). A systemic review on the synthesis, characterization and applications of palladium nanoparticles in biomedicine. Appl. Biochem. Biotechnol..

[12] Zhang M., Song W., Tang Y., Xu X., Huang Y., Yu D. (2022). Polymer-based nanofiber–nanoparticle hybrids and their medical applications. Polymers.

[13] Soares P.I., Borges J.P. (2021). Recent advances in magnetic electrospun nanofibers for cancer theranostics application. Prog. Nat. Sci. Mater. Int..

[14] Bustamante-Torres M., Romero-Fierro D., Estrella-Nuñez J., Arcentales-Vera B., Chichande-Proaño E., Bucio E. (2022). Polymeric composite of magnetite iron oxide nanoparticles and their application in biomedicine: A review. Polymers.

[15] Kush P., Kumar P., Singh R., Kaushik A. (2021). Aspects of high-performance and bio-acceptable magnetic nanoparticles for biomedical application. Asian J. Pharm. Sci..

[16] Yilmaz N.D. (2018). Smart Textiles: Wearable Nanotechnology.

[17] Barros L.N.L.C., Araujo R.N.D., Nascimento E.P.D., Gama A.J.D.A., Neves G.A., Torres M.A.M., Menezes R.R. (2024). Influence of fast drying on the morphology of α-Fe_2_O_3_ and FeMnO_3_/α-Fe_2_O_3_ fibers produced by solution blow spinning. Nanomaterials.

[18] Zhang H., Xia J., Pang X., Zhao M., Wang B., Yang L., Fu S. (2017). Magnetic nanoparticle-loaded electrospun polymeric nanofibers for tissue engineering. Mater. Sci. Eng. C.

[19] Oun A.A., Shankar S., Rhim J.W. (2020). Multifunctional nanocellulose/metal and metal oxide nanoparticle hybrid nanomaterials. Crit. Rev. Food Sci. Nutr..

[20] Zadehnazari A. (2023). Metal oxide/polymer nanocomposites: A review on recent advances in fabrication and applications. Polym. Plast. Technol. Mater..

[21] Garcia M.F., Neves G.A., Nascimento E.P., Loureiro F.J., Araújo A.J., Raimundo R.A., Menezes R.R. (2024). Solution blow spinning of Co_3_O_4_ nanofibers as electrocatalyst for oxygen evolution processes. Ceram. Int..

[22] Al Kayal T., Giuntoli G., Cavallo A., Pisani A., Mazzetti P., Fonnesu R., Losi P. (2023). Incorporation of copper nanoparticles on electrospun polyurethane membrane fibers by a spray method. Molecules.

[23] Abdullah J.A.A., Perez-Puyana V., Guerrero A., Romero A. (2023). Novel hybrid electrospun poly (ε-caprolactone) nanofibers containing green and chemical magnetic iron oxide nanoparticles. J. Appl. Polym. Sci..

[24] Sawant S.R., Kalla S., Murthy Z.V.P. (2023). Nanomaterial-incorporated membrane distillation membranes: Characteristics, fabrication techniques and applications. Chem. Eng. Technol..

[25] Voorhis C., González-Benito J., Kramar A. (2024). “Nano in Nano”—Incorporation of ZnO Nanoparticles into Cellulose Acetate–Poly (Ethylene Oxide) Composite Nanofibers Using Solution Blow Spinning. Polymers.

[26] Kakoria A., Sinha-Ray S. (2018). A Review on biopolymer-based fibers via electrospinning and solution blowing and their applications. Fibers.

[27] Kramar A., González-Benito J. (2023). Preparation of cellulose acetate film with dual hydrophobic-hydrophilic properties using solution blow spinning. Mater. Des..

[28] Tsinas Z., Tao R., Forster A.L. (2021). Solution blow spinning of polymeric nano-composite fibers for personal protective equipment. J. Vis. Exp..

[29] Oliveira J.E., Mattoso L.H.C., Orts W.J., Medeiros E.S. (2013). Structural and Morphological Characterization of Micro and Nanofibers Produced by Electrospinning and Solution Blow Spinning: A Comparative Study. Adv. Mater. Sci. Eng..

[30] Scaffaro R., Settanni L., Gulino E.F. (2023). Release Profiles of Carvacrol or Chlorhexidine of Pla/Graphene Nanoplatelets Membranes Prepared Using Electrospinning and Solution Blow Spinning: A Comparative Study. Molecules.

[31] Vilches J.L., Filho M.D.S.M.D.S., Rosa M.D.F., Sanches A.O., Malmonge J.A. (2019). Fabrication of fish gelatin microfibrous mats by solution blow spinning. Mater. Res..

[32] Eticha A.K., Akgul Y. (2024). Optimization of gelatin nanofibrous webs fabricated via electrically assisted solution blow spinning for air filtration applications. Int. J. Sci. Environ. Technol..

[33] Sardareh E.A., Shahzeidi M., Ardestani M.T.A., Mousavi-Khattat M., Zarepour A., Zarrabi A. (2022). Antimicrobial activity of blow spun PLA/gelatin nanofibers containing green synthesized silver nanoparticles against wound infection-causing bacteria. Bioengineering.

[34] Kołodziejek D., Łopianiak I., Tadko O., Drozd M., Wojasiński M., Jastrzębska E. (2023). Magnetic polyurethane nanomaterials: A novel approach for in vitro cardiac cell maturation and culture. Polym. Test.

[35] Khdary N.H., Almuarqab B.T., El Enany G. (2023). Nanoparticle-embedded polymers and their applications: A review. Membranes.

[36] Araujo R.N., Nascimento E.P., Raimundo R.A., Macedo D.A., Mastelaro V.R., Neves G.A., Menezes R.R. (2021). Hybrid hematite/calcium ferrite fibers by solution blow spinning: Microstructural, optical and magnetic characterization. Ceram. Int..

[37] Selvaraj S., Thangam R., Fathima N.N. (2018). Electrospinning of casein nanofibers with silver nanoparticles for potential biomedical applications. Int. J. Biol. Macromol..

[38] Kowsalya E., MosaChristas K., Balashanmugam P., Rani J.C. (2019). Biocompatible silver nanoparticles/poly (vinyl alcohol) electrospun nanofibers for potential antimicrobial food packaging applications. Food Packag. Shelf Life.

[39] Aamer H., Kim S.B., Oh J.M., Park H., Jo Y.M. (2021). ZnO-impregnated polyacrylonitrile nanofiber filters against various phases of air pollutants. Nanomaterials.

[40] Berret J.-F., Sandre O., Mauger A. (2007). Size Distribution of superparamagnetic particles determined by magnetic sedimentation. Langmuir.

[41] Safi M., Courtois J., Seigneuret M., Conjeaud H., Berret J.F. (2011). The effects of aggregation and protein corona on the cellular internalization of iron oxide nanoparticles. Biomaterials.

[42] Vitorazi L., Berret J.F. (2024). Mixing order asymmetry in nanoparticle-polymer complexation and precipitation revealed by isothermal titration calorimetry. J. Phys. Chem. B.

[43] Sun Y., Ma M., Zhang Y., Gu N. (2004). Synthesis of Nanometer-Size Maghemite Particles from Magnetite. Colloids Surf. A Physicochem. Eng. Asp..

[44] Bee A., Massart R., Neveu S. (1995). Synthesis of very fine maghemite particles. J. Magn. Magn. Mater..

[45] Liang Q., Zhou C., Rehman A., Qayum A., Liu Y., Ren X. (2023). Improvement of physicochemical properties, microstructure and stability of lotus root starch/xanthan gum stabilized emulsion by multi-frequency power ultrasound. Ultrason. Sonochem..

[46] Pochapski D.J., Santos C.C., Leite G.W., Pulcinelli S.H., Santilli C.V. (2021). Zeta potential and colloidal stability predictions for inorganic nanoparticle dispersions: Effects of experimental conditions and electrokinetic models on the interpretation of results. Langmuir.

[47] Fresnais J., Yan M., Courtois J., Bostelmann T., Bée A., Berret J.F. (2013). Poly(acrylic acid)-coated iron oxide nanoparticles: Quantitative evaluation of the coating properties and applications for the removal of a pollutant dye. J. Colloid Interface Sci..

[48] Dadfar S.M., Camozzi D., Darguzyte M., Roemhild K., Varvarà P., Metselaar J., Lammers T. (2020). Size-isolation of superparamagnetic iron oxide nanoparticles improves MRI, MPI and hyperthermia performance. J. Nanobiotechnol..

[49] Guibert C., Dupuis V., Fresnais J., Peyre V. (2015). Controlling nanoparticles dispersion in ionic liquids by tuning the pH. J. Colloid Interface Sci..

[50] Yan M., Fresnais J., Berret J.F. (2010). Growth mechanism of nanostructured superparamagnetic rods obtained by electrostatic co-Assembly. Soft Matter.

[51] Bandhu A., Sutradhar S., Mukherjee S., Greneche J.M., Chakrabarti P.K. (2015). Synthesis, characterization and magnetic property of maghemite (γ-Fe_2_O_3_) nanoparticles and their protective coating with pepsin for bio-functionalization. Mater. Res. Bull..

[52] Shayan N.N., Mirzayi B. (2015). Adsorption and removal of asphaltene using synthesized maghemite and hematite nanoparticles. Energy Fuels.

[53] Silverstein R.M., Webster F.X., Kiemle D.J. (2005). Spectrometric Identification of Organic Compounds.

[54] Campos E.A., Pinto D.V., Oliveira J.I., Mattos E.D., Dutra R.D. (2015). Synthesis, characterization and applications of iron oxide nanoparticles-a short review. J. Aerosp. Technol. Manag..

[55] Alves A.F., Mendo S.G., Ferreira L.P., Mendonça M.H., Ferreira P., Godinho M., Cruz M.M., Carvalho M.D. (2016). Gelatine-assisted synthesis of magnetite nanoparticles for magnetic hyperthermia. J. Nanopart. Res..

[56] Bhavsar V., Tripathi D. (2017). Structural, optical and aging studies of biocompatible PVC-PVP blend films. J. Polym. Eng..

[57] Winsett J., Moilanen A., Paudel K., Kamali S., Ding K., Cribb W., Seifu D., Neupane S. (2019). Quantitative determination of magnetite and maghemite in iron oxide nanoparticles using Mössbauer spectroscopy. Disco Appl. Sci..

[58] Dar M.I., Shivashankar S.A. (2014). Single crystalline magnetite, maghemite and hematite nanoparticles with rich coercivity. RSC Adv..

[59] Koczkur K.M., Mourdikoudis S., Polavarapu L., Skrabalak S.E. (2015). Polyvinylpyrrolidone (PVP) in nanoparticle synthesis. Dalton Trans..

[60] Das M.P., Suguna P.R., Prasad K.A., Vijaylakshmi J.V., Renuka M. (2017). Extraction and characterization of gelatin: A functional biopolymer. Int. J. Pharm. Pharm. Sci..

[61] Ahmad T.A.T., Ismail A.I., Ahmad S.A., Khalil K.A., Awad E.A.E., Leo T.K., Imlan J.C., Sazili A.Q. (2018). Characterization of gelatin from bovine skin extracted using ultrasound subsequent to bromelain pretreatment. Food Hydrocoll..

[62] Mishra R., Varshney R., Das N., Sircar D., Roy P. (2019). Synthesis and characterization of gelatin-PVP polymer composite scaffold for potential application in bone tissue engineering. Eur. Polym. J..

[63] Salles T.H.C., Lombello C.B., D’ávila M.A. (2015). Electrospinning of Gelatin/Poly(Vinyl Pyrrolidone) Blends from Water/Acetic Acid Solutions. Mater. Res..

