# Supplementary material for: Magnetic Nanoparticles in Biopolymer Fibers: Fabrication Techniques and Characterization Methods
Source: Polymers (Basel). 2024 Oct 3;16(19):2805. doi: 10.3390/polym16192805 (PMC11478581; doi:10.3390/polym16192805)
Supplement: Supplementary file 1 [file polymers-16-02805-s001.zip › polymers-3118677-supplementary.pdf]

Outline

S1 – Qualitative demonstration of the magnetic responsiveness of γ-Fe_2O_3-NPs.

S2 – List of all reagents and solvents used in studies

S3 – The behavior of nanoparticles as a function of the pH of the medium

S4 – Composition and preparation methods of solutions used in the spinning process.

Supporting information S1

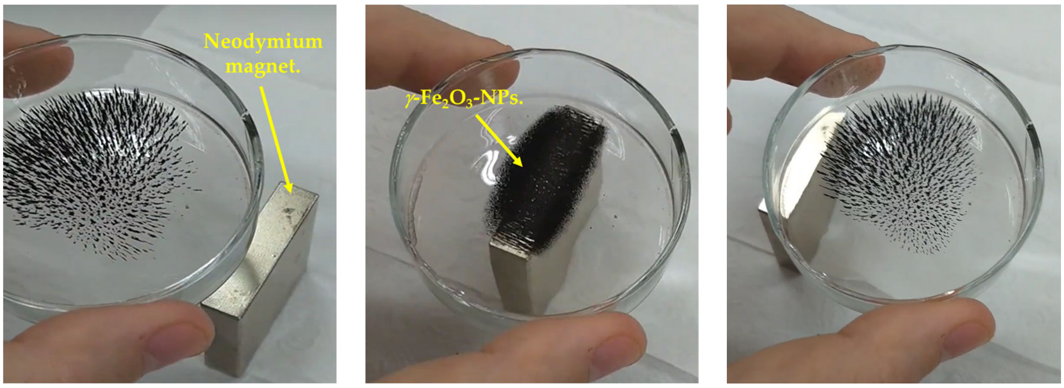

(a) (b) (c)

Figure S1. Qualitative demonstration of the magnetic responsiveness of γ-Fe_2O_3-NPs: (a) γ-Fe_2O_3-NPs positioned to the left of a neodymium magnet, (b) γ-Fe_2O_3-NPs directly above the neodymium magnet, and (c) γ-Fe_2O_3-NPs positioned to the right of the neodymium magnet.

Supporting Information S2

List of all reagents and solvents used in studies

Table S1. List of all chemicals employed in the synthesis and coating or fibers formation.

**Experimental step**	**Reagents and solutions**
Synthesis of iron oxide nanoparticles	- Ferric chloride hexahydrate ($FeCl_3.6H_2O$) – Analytical grade, Synth
	- Ferrous chloride tetrahydrate ($FeCl_2.4H_2O$) – Analytical grade, Sigma-Aldrich
	- Sodium citrate dihydrate ($Na_3C_6H_5O_7.2H_2O$) – Analytical grade, Synth
	- Ferric nitrate nonahydrate ($FeNO_3.9H_2O$) – Analytical grade, Synth
	- Ammonium hydroxide 28 – 30 % (NH_4OH) –Analytical grade, Vetec
	- Nitric acid 65 % (HNO_3) – Analytical grade, Vetec
Coating of iron oxide particles	- Iron oxide nanoparticles in aqueous dispersion
	- Poly(acrylic acid) – PAA, with a molecular weight of 2000 $g.mol^{-1}$, Sigma-Aldrich
	- HNO_3 65 % – Analytical grade, Vetec
	- NH_4OH 28 – 30 % – Analytical grade, Vetec
Solution blow spinning for fiber formation	- Hexane ($CH_3(CH_2)_4CH_3$) – Analytical grade, Neon
	- Chloroform ($CHCl_3$) – Analytical grade, Synth
	- Acetone ($(CH_3)_2CO$) – Analytical grade, Synth
	- Isopropyl alcohol ($CH_3CHOHCH_3$) – Analytical grade, Synth
	- Ethyl alcohol (C_2H_6O) – Analytical grade, Dinâmica
	- Methyl alcohol (CH_3OH) – Commercial, Synth
	- Glacial acetic acid (CH_3COOH) – Commercial, Synth
	- Bovine skin gelatin type B – Sigma-Aldrich
	- Poly(vinylpyrrolidone) – PVP, 360,000 $g.mol^{-1}$ – Sigma-Aldrich
	- Iron oxide nanoparticles coated with PAA

Supporting Information S3

The behavior of nanoparticles as a function of the pH of the medium

To verify the stability of nanoparticles in the aqueous medium, a determined amount of water at different pH values (1.00 mL) was placed in labeled glass vials, and up to 50 µL of either synthesized or coated NPs in water dispersion were added. The behavior of the mixtures was observed for 30 min. The results are shown in Figure S2. It was observed that before coating the nanoparticles containing citrate) are stable at pH ≤ 2 and after coating with PAA_{2k}, γ-Fe_2O_3-NPs-PAA_{2k} are stable at approximately pH ≥ 4. These characteristics indicate the success of the coating.

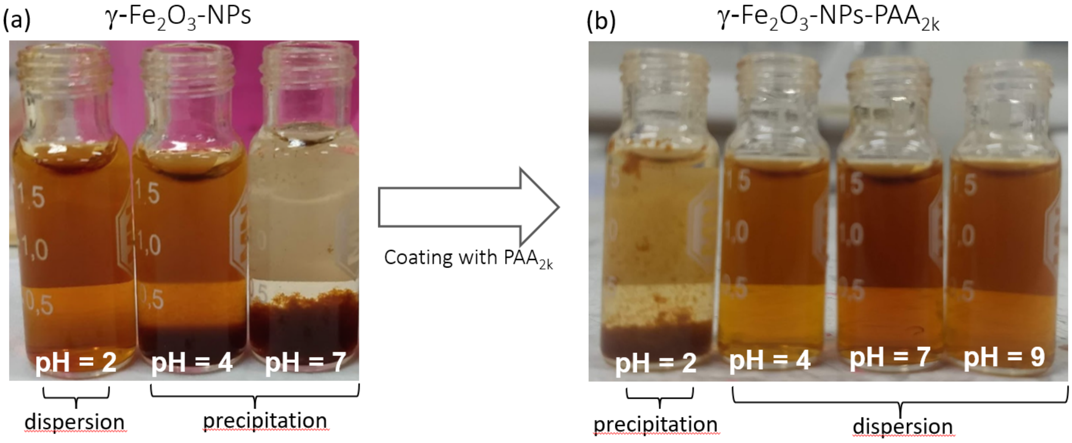

Figure S2. Images of vials containing the nanoparticles γ-Fe_2O_3-NPs (R = 0.25 %) in aqueous medium pH 2 – 7 (a) and γ-Fe_2O_3-NPs-PAA_{2k} NPs (R = 0.25%) in aqueous medium pH 2 -9.

Supporting Information S4

Composition and preparation methods of solutions used in the spinning process.

Several polymeric solutions were prepared for the test in the spinning system to evaluate effective fiber formation and solvent evaporation, the quality of formed fibers, and processing parameters for subsequent NPs incorporation. All solutions were prepared by dissolving the polymers in acetic acid, with gelatin dissolved first, followed by PVP, and then adding methanol or ethanol to the mixture. For NPs incorporation into the polymeric solution, solutions with 2.50% w/v gelatin and 6.00% w/v PVP were prepared in a mixture of 95% acetic acid (in water) and methanol (solvent ratio of 3:1 acetic acid to methanol). The solutions were prepared: the required gelatin mass was weighed and dissolved in acetic acid with magnetic stirring. After complete gelatin dissolution, the required mass of PVP and half the required volume of methanol were added, and the mixture was stirred until complete dissolution. The coated particles in aqueous solution were separately dispersed in the remaining volume of methanol, and this mixture was slowly added to the polymeric solution in 100 µL aliquots with mechanical homogenization between each addition. Table S2 presents a representative list ofsolutions prepared for gelatin and PVP testing.

Table S2. The composition of polymeric solutions used for fiber fabrication at SBS procedure.

	Gelatin (% m/v)	PVP (% m/v)	Solvent 1	Solvent 2	Ratio Solv. 1 : Solv. 2
**S1**	-	7.0	CH_3COOH	CH_3CH_2OH	1 : 4
**S2**	3.0	5.0	CH_3COOH	CH_3OH	1 : 3
**S3**	-	7.0	CH_3COOH	CH_3OH	3 : 1
**S4**	3.0	5.0	CH_3COOH	CH_3OH	3 : 1
**S5**	3.0	6.0	CH_3COOH	CH_3OH	3 : 1
**S6**	3.0	6.0	CH_3COOH	CH_3CH_2OH	3 : 1
**S7**	3.0	7.0	CH_3COOH	CH_3OH	3 : 1
**S8**	3.0	6.0	CH_3COOH 97 % *	CH_3OH	3 : 1
**S9**	3.0	6.0	CH_3COOH 93 %	CH_3OH	3 : 1
**S10**	2.5	6.0	CH_3COOH	CH_3CH_2OH	3 : 1
**S11**	2.5	6.0	CH_3COOH 93 %	CH_3OH	3 : 1

**S12**	2.5	6.0	CH_3COOH 95 %	CH_3OH	3 : 1
**S13**	2.5	6.0	CH_3COOH 93 %	CH_3OH	3 : 2
**S14**	3.0	6.0	CH_3COOH	CH_3OH	4 : 1
**S15**	2.5	6.0	CH_3COOH 93 %	CH_3OH	4 : 1
**S16**	3.0	6.0	CH_3COOH	CH_3OH	5 : 1
**S17**	3.0	6.0	CH_3COOH 93 %	CH_3OH	7 : 2
**S18**	3.0	6.0	CH_3COOH 93 %	CH_3CH_2OH	7 : 2
**S19**	3.0	6.0	CH_3COOH	CH_3OH	7 : 3
**S20**	2.5	6.0	CH_3COOH 95 %	CH_3OH	7 : 3

*Concentration of acetic acid (% v/v in water) diluted from commercial reagent (with 99.5% purity, according to the manufacturer).

Microscopic images of fibers obtained from some of the solutions listed in Table S2 are shown in Figure S3. The images presented in Figure S3 represent characterized fibers with defects. In Figure S3 (a), the fiber obtained from mixture S1 is adhered to the substrate and presents beads. The fibers obtained from mixtures S3 and S12 present irregular diameter distribution, and fibers obtained from mixtures S6, S10, and S11 present beads, irregular distribution, and irregularities on the fiber surface. It is important to highlight that not all solutions yielded collectable fibers, therefore not all combinations listed are represented by a microscopic image. When fibers were not collected and characterized via SEM, one of the following reasons was observed for the sample: it wasn't possible to produce fibers despite the adjusts in spinning parameters; the fibers were formed, however there were visible drops of solvent (beads) in the fibers collected; the fibers were light and disperse, even though they were directed to the collector, and it wasn't possible to collect them; the fibers were collected however it wasn't possible to remove them from the aluminum foil substrate due to the presence of remaining solvent.

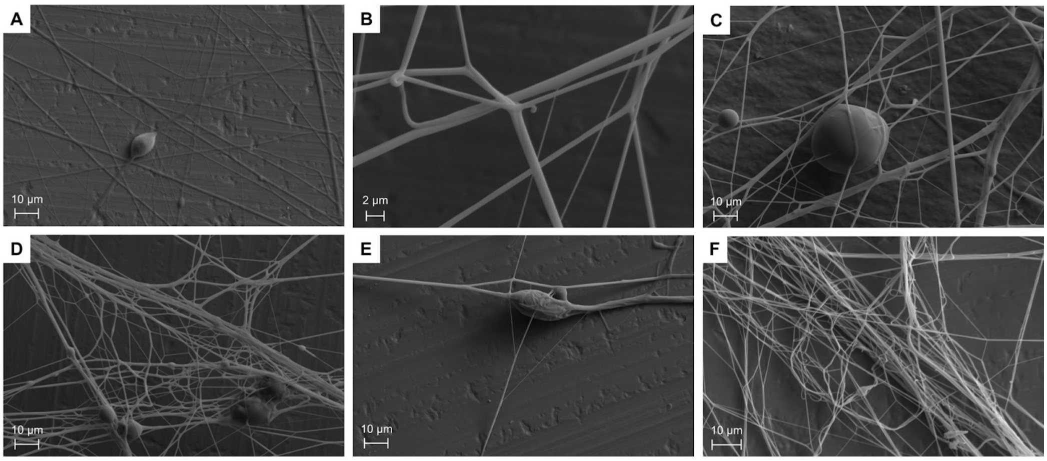

Figure S3: SEM images of fibers collected from mixtures A) S1, B) S3, C) S6, D) S10, E) S11 and F) S12.
